# Drug Vulnerabilities and Disease Prognosis Linked to the Stem Cell-Like Gene Expression Program Triggered by the RHO GTPase Activator VAV2 in Hyperplastic Keratinocytes and Head and Neck Cancer

**DOI:** 10.3390/cancers12092498

**Published:** 2020-09-03

**Authors:** Luis Francisco Lorenzo-Martín, Mauricio Menacho-Márquez, Xosé R. Bustelo

**Affiliations:** 1 Centro de Investigación del Cáncer, CSIC-University of Salamanca, 37007 Salamanca, Spain; mmenacho@conicet.gov.ar; 2 Instituto de Biología Molecular y Celular del Cáncer, CSIC-University of Salamanca, 37007 Salamanca, Spain; 3 Centro de Investigación Biomédica en Red de Cáncer (CIBERONC), CSIC-University of Salamanca, 37007 Salamanca, Spain

**Keywords:** VAV2, VAV family, Rac1, RhoA, GTPases, skin, head and neck, keratinocytes, cancer, oncogenes, signaling, regenerative proliferation, stem cells

## Abstract

**Simple Summary:**

Head and neck squamous cell carcinoma are epithelial tumors with a very poor prognosis. They are also in high need of new targeted and immune-based therapeutics to limit tumor recurrence and improve long-term survival. The poor prognosis of patients with head and neck tumors is usually associated with histological features associated with poor differentiation and high proliferative activity found in their tumor biopsies. Therefore, it is of paramount importance to identify vulnerabilities associated with such pathobiological programs. In this work, the authors utilize a stem cell-like program linked to the deregulated activity of VAV2, a protein frequently overexpressed in this type of tumors, to identify new therapeutic targets that can discriminate tumors from healthy cells. The authors also show that this gene expression program can be used to stratify patients according to long-term prognosis.

**Abstract:**

We have recently shown that VAV2, a guanosine nucleotide exchange factor that catalyzes the stimulation step of RHO GTPases, is involved in a stem cell-like (SCL) regenerative proliferation program that is important for the development and subsequent maintenance of the tumorigenesis of both cutaneous (cSCC) and head and neck squamous cell carcinomas (hnSCC). In line with this, we have observed that the levels of the *VAV2* mRNA and VAV2-regulated gene signatures are associated with poor prognosis in the case of human papillomavirus-negative hnSCC patients. These results suggest that the SCL program elicited by VAV2 in those cells can harbor therapeutically actionable downstream targets. We have addressed this issue using a combination of both in silico and wet-lab approaches. Here, we show that the VAV2-regulated SCL program does harbor a number of cell cycle- and signaling-related kinases that are essential for the viability of undifferentiated keratinocytes and hnSCC patient-derived cells endowed with high levels of VAV2 activity. Our results also show that the VAV2-regulated SCL gene signature is associated with poor hnSCC patient prognosis. Collectively, these data underscore the critical role of this VAV2-regulated SCL program for the viability of both preneoplastic and fully transformed keratinocytes.

## 1. Introduction

One of the hallmarks of cancer cells is their ability to hijack molecular programs that are usually restricted to progenitor and stem cells [1]. This provides cancers with unrestrained growth potential, plasticity, adaptability to environmental changes, and survival mechanisms against many of the current therapies [1]. In line with this, the enrichment of stem cell-like features in tumors is frequently one of the leading parameters associated with worse prognosis. This is clearly exemplified in squamous cell carcinomas (SCCs), in which the presence of such features correlate with poor patient survival [2]. As a consequence, there is a growing interest in the blockade of the stem cell-like features of tumor cells [3]. Nevertheless, doing so without negatively affecting the physiological function of healthy stem cells remains a major concern when considering clinical applications [1]. As it occurs in the case of other cancer-associated pathobiological programs, the eventual success of such therapies would rely on the degree of addiction that tumor cells might develop for the targeted processes when compared to the healthy counterparts [4]. In this context, the identification of biomarkers able to predict tumor cell dependency towards specific stem cell-associated molecular pathways holds great translational potential.

Recent sequencing and molecular taxonomy studies have revealed that the activation of RHO GTPase-regulated biological processes is a common signaling feature for most SCCs [5,6]. Consistent with this, we have recently found that VAV2, a guanosine nucleotide exchange factor (GEF) that promotes the activation of RHO GTPases during cell signaling [7,8,9,10], plays a pivotal role in this process. Thus, its chronic activation in mice leads to epithelial hyperplasia in the skin, palate, and other head and neck areas. The same phenotype is observed when tested in three-dimensional (3D) organotypic cultures. Although not enough for triggering cell transformation per se, we also observed that its constitutive activation in the skin epidermis creates a pro-tumorigenic niche that, upon the generation of further genetic lesions, favors enhanced tumor formation and progression in mice [11]. Conversely, the use of catalytically hypomorphic *Vav2* knock-in mice has revealed that the enzymatic activity of this GEF is essential for skin SCC initiation, promotion and progression [12]. Perhaps more importantly, we have observed that the elimination of endogenous VAV2 eliminates the tumorigenic potential of hnSCC patient-derived cells and standard hnSCC cancer cell lines using both orthotopic xenotransplants in vivo and 3D organotypic cultures [11]. Further supporting the relevance of this GEF for this type of tumors, we have found that the levels of the *VAV2* mRNA and VAV2-regulated gene signatures correlate with poor prognosis in the case of human papilloma virus-negative hnSCC patients [11].

It is as yet unclear whether the foregoing data can be exploited pharmacologically to treat cSCC and hnSCC patients given the difficulties traditionally found in the development of high-affinity inhibitors for most RHO GEFs [6]. An alternative avenue to achieve this goal is to find therapeutic vulnerabilities among the VAV2-regulated gene expression programs present in epithelial cells. One of them is a SCL program associated with regenerative proliferation and extensive cell undifferentiation driven by the VAV2-mediated activation of the transcriptional factors c-MYC and YAP/TAZ. Consistent with this, we have found that c-MYC and YAP/TEAD inhibitors lead to reduced proliferation and extensive differentiation of keratinocytes ectopically expressing an active version of VAV2, respectively [11].

To explore this possibility, we implemented here a multifaceted approach aimed at the identification of therapeutic Achilles’ heels in the VAV2-regulated SCL program. This has resulted in the identification of a number of cell cycle- and signaling-regulated protein kinases that are essential for the viability of undifferentiated cells associated with upregulated VAV2 signaling. Interestingly, we have found that the inhibition of these kinases is not detrimental for normal epithelial cells at the concentrations used in our experiments. Finally, we demonstrate that the VAV2-driven SCL program is associated with hnSCC patient survival. Collectively, this work indicates that the VAV2-regulated SCL programs can be used both as therapeutic and diagnostic tools for cSCC and hnSCC.

## 2. Methods

### 2.1. Ethics Statement

Animal work was done according to protocols approved by the Bioethics committee of Salamanca University and the Castilla y León autonomous government (Approval Number 315).

### 2.2. In Silico Analyses

*Vav2*^Onc^ mouse skin expression microarray data were obtained from the GSE124019 dataset generated in a previous work [11]. Signal intensity values were obtained from CEL files after applying the Robust Multichip Average (RMA) function from the ‘affy’ package for background adjustment, quantile normalization and summarization [13]. Gene Set Enrichment Analyses (GSEA) were carried out with the described gene sets using gene set permutations (*n* = 1000) for the assessment of significance and signal-to-noise metric for ranking genes [14]. The stemness-related gene sets used in GSEA analyses include those for epidermal progenitor cells [15], SCC initiating cells [16], and normal embryonic stem cells [17,18,19].

Functional annotation was performed using Metascape (accessed on September 2019) [20]. Known functional interactions among relevant genes were obtained through the String tool [21]. Cytoscape open-source software was used to perform network data integration and visualization [22].

For the discovery of transcription factor binding motifs in the promoters of the co-regulated genes, the iRegulon open-source software was used [23]. A collection of 9713 position weight matrices (PWMs) was applied to analyze 10 kb centered around the TSS. With a maximum false discovery rate (FDR) on motif similarity below 0.001, we performed motif detection, track discovery, motif-to-factor mapping and target detection.

Prediction of drug vulnerability and gene dependency were performed using LINCS [24] and DepMap [25], respectively. ssGSEA [14,26] against the GSE30784 dataset [27] was used to evaluate the enrichment of the *Vav2*^Onc^-driven stemness signature in SCC tumors as indicated elsewhere [11].

Overall survival analyses were performed through Kaplan–Meier estimates according to the enrichment of the *Vav2*^Onc^-regulated stemness signature using the GSE41613 dataset [28]. The median of the enrichment distribution for the signature was used to establish the low and high expression groups and, subsequently, the Mantel–Cox test was applied to statistically validate the differences between the survival distributions.

### 2.3. Primary Mouse Keratinocytes

Primary keratinocytes were isolated from newborn *Vav2*^Onc/Onc^ and control animals [29] as described elsewhere [30]. To this end, the skin were extracted from mice of the indicated genotypes, treated with dispase (250 U/mL; Roche, Basel, Switzerland; Catalog No. 04942078001) overnight at 4 °C. The next day, the epidermis was separated from the dermis and treated with Accutase (CELLnTEC, Bern, Switzerland; Catalog No. CnT-Accutase-100) for 30 min at 37 °C to extract the keratinocytes. Cultures were them kept in CnT07 medium (CELLnTEC, Catalog No. CnT-07) on type I collagen-coated plates (BD Biosciences, San Jose, CA, USA; Catalog No. 356400).

### 2.4. Human Keratinocyte Cell Lines

Immortalized, primary neonatal human keratinocytes (KerCT cells) were obtained from the American Type Culture Collection (ATCC, Manassas, VA, USA; Catalog No. CRL-4048). These cells have been immortalized by ectopically expressing human TERT and CDK4. KerCT cell derivatives ectopically expressing mouse Vav2^Onc^ were described before [11]. All these cells were cultured in KGM-Gold medium (Lonza, Basel, Switzerland; Catalog No. 00192060).

SCC-25 cells, obtained from Dr. Salvador A. Benitah (Institute for Research in Biomedicine, Barcelona, Spain), were cultured in KSFM medium (Thermo Fisher Scientific, Waltham, MA, USA; Catalog No. 17005-042) supplemented with 25 µg/mL bovine pituitary extract (Thermo Fisher Scientific, Catalog No. 17005-042) and 0.5 ng/mL human epidermal growth factor (Thermo Fisher Scientific, Catalog No. 17005-042).

hnSCC (tongue-located) patient-derived cells were provided by Dr. Salvador A. Benitah [31]. VdH15 cells were cultured in KSFM medium supplemented with 25 µg/mL bovine pituitary extract and 0.5 ng/mL human epidermal growth factor (Thermo Fisher Scientific, Catalog No. 17005-042). VdH01 cells were cultured in a FAD^+^ medium that was composed of three parts of DMEM (Gibco, Waltham, MA, USA; Catalog No. 21969) and one part of Ham’s F12 medium (Thermo Fisher Scientific, Catalog No.11765054) supplemented with 10% fetal bovine serum (Gibco, Catalog No. 10270106), 1.8 × 10^–4^ M adenine (Sigma-Aldrich, Saint Louis, MO, USA Catalog No. A2786-5G), 0.5 µg/mL hydrocortisone (Sigma-Aldrich, Saint Louis, MO, USA; Catalog No. H4001-1G), 5 µg/mL insulin (Thermo Fisher Scientific, Catalog No. 12585014), 10 ng/mL EGF (PreproTech, Rocky Hill, NJ, USA; Catalog No. AF-100-15), 10^–10^ M cholera toxin (Sigma-Aldrich, Catalog No. C8052-5MG), and 2 mM L-glutamine (Gibco, Catalog No. 25030024).

### 2.5. Three-Dimensional Organotypic Cultures

Exponentially growing keratinocytes maintained in CnT-Prime medium (CELLnTEC, Catalog No. CnT-PR) were detached using Accutase, centrifuged at 300× *g* for 5 min at room temperature, and counted. A total of 2 × 10^5^ cells were then seeded onto polycarbonate inserts (Thermo Fisher Scientific, Catalog No. 140620) and cultured for two days in CnT-Prime medium. Upon confluency, the medium was changed to 3D-Barrier (CELLnTEC, Catalog No. CnT-PR-3D) and the air-lift performed according to the manufacturer’s instructions. 3D cultures were maintained for 12 days with three medium changes per week. When indicated, the inhibitors and corresponding vehicles were applied in the sixth day after carrying out the airlift and refreshed with the medium changes indicated above. Inhibitors used included Alisertib (Selleckchem, Munich, Germany; Catalog No. S1133), XL413 (Selleckchem, Catalog No. S7547), AZD7762 (Selleckchem, Catalog No. S1532), and BI2536 (Selleckchem, Catalog No. S1109) (10 nM each). Concentrations of inhibitors were selected based on the induction of minor effect in the organotypic structures formed by control cells.

### 2.6. Histological Studies

Three-dimensional cultures were fixed in 4% paraformaldehyde, paraffin embedded, cut in 2–3 µm thick sections, and stained with hematoxylin-eosin. Images were captured using an Olympus BX51 microscope coupled to an Olympus DP70 digital camera. The thickness of the epidermal layer and/or total skin thickness were measured in vertical cross-sections using the ImageJ software (NIH, Bethesda, MD, USA) [32].

### 2.7. Statistics

The number of biological replicates (*n*), the type of statistical tests performed, and the statistical significance are indicated for each experiment either in the figure legends or in the main text. Data normality and equality of variances were analyzed with Shapiro-Wilk and Bartlett’s tests, respectively. Parametric distributions were analyzed using Student’s *t*-tests (when comparing two experimental groups) or ANOVA followed by Tukey’s HSD tests (when comparing more than two experimental groups with every other group). In all cases, values were considered significant when *p* ≤ 0.05. Data obtained are given as the mean ± SEM.

## 3. Results

### 3.1. Discovery of Pharmacological Targets in the Vav2^Onc^-Driven SCL Keratinocyte Program

**Figure 1:**
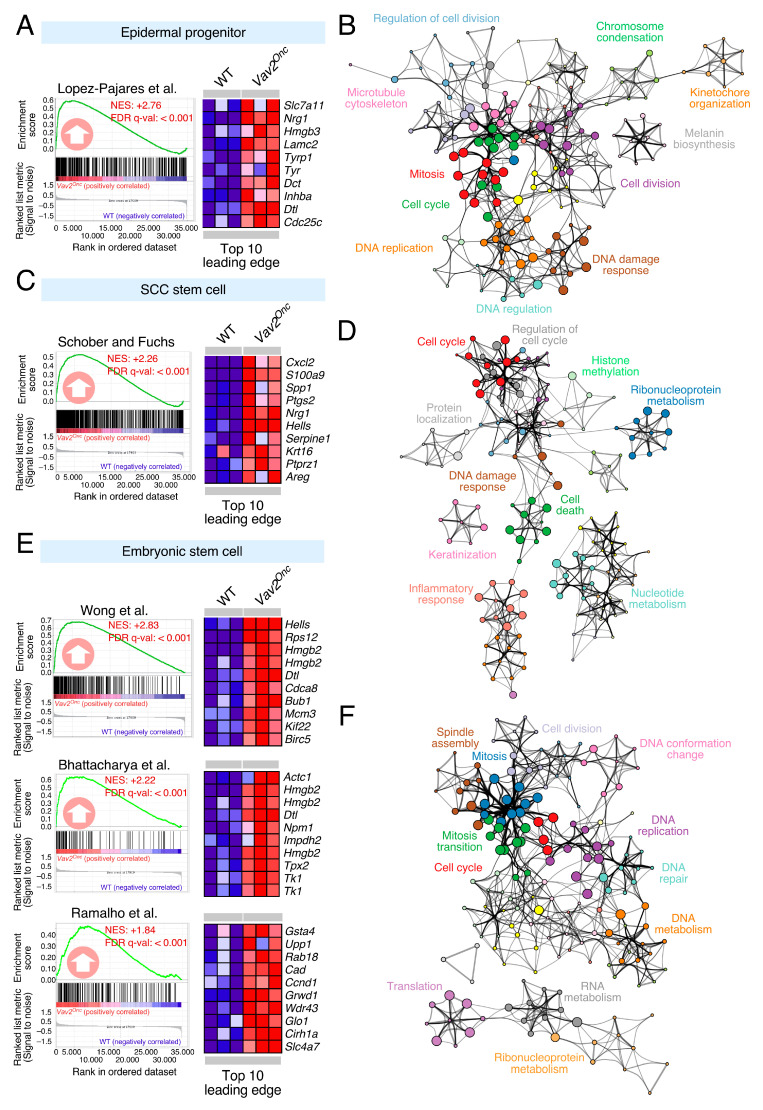
VAV2 controls a stem cell-like transcriptional program in keratinocytes. (**A**,**C**,**E**) Gene Set Enrichment Analyses (GSEA) of the indicated gene sets (top) in the *Vav2^Onc^*-dependent skin transcriptome. The normalized enrichment scores (NES) and false discovery rate *q*-values (FDR *q*-val) are indicated within each graph. Positive enrichments are indicated with upward-pointing arrows. The expression heatmap for the top 10 leading-edge probesets is also included. (**B**,**D**,**F**) Functional networks depicting the most enriched functional terms in the leading edge of the GSEA shown in A,C,E. Node size is proportional to the significance of the enrichment. Related functional terms are linked by edges.

In order to understand the role of upregulated VAV2 signaling in epithelial cells, we recently utilized a catalytic gain-of-function knock-in mouse strain that expresses a mutant version of the mouse Vav2 protein (N-terminal 1–186 deletion, referred to hereafter as Vav2^Onc^). This mutant protein exhibits constitutive GEF activity due to the removal of most N-terminal autoinhibitory domains that maintain the inactive state of VAV family proteins in the absence of tyrosine phosphorylation [7,8,10]. Due to this, the biological activity of this active version must reflect the output of the wild-type protein under optimal tyrosine phosphorylation conditions according to the current VAV family regulatory model [7,8,10]. Importantly, Vav2^Onc^ is expressed from the endogenous locus and, therefore, exhibits pattern and levels of expression in tissues identical to the endogenous wild-type counterpart [12]. These mice develop extensive hyperplasia in the epidermal linings of both the skin and head and neck areas [11]. Microarray and functional studies revealed that this phenotype develops as the consequence of the activation of a regenerative proliferation program that contains molecular features typical of an undifferentiated, SCL state [11]. In agreement with these previous studies, Gene Set Enrichment Analyses (GSEA) revealed that the Vav2^Onc^-regulated transcriptome present in mouse skin epithelial cells harbors gene signatures previously found in epidermal progenitor cells, SCC initiating cells, and normal embryonic stem cells (Figure 1A,C,E). This SCL state is highly aberrant, since it does show any statistically significant enrichment in gene signatures corresponding to the transcriptomes of hair follicle stem cells.

The functional annotation of these Vav2^Onc^-regulated SCL gene programs revealed quite similar functional features. Thus, the three signatures were enriched in proliferation-associated genes such as those involved in cell cycle regulation, chromosome condensation, DNA replication, spindle assembly, mitosis, cell division or anabolic processes (Figure 1B,D,F). In the case of the SCC initiating cell-like signature, this enrichment also includes genes linked to inflammatory, cell death, and histone methylation responses (Figure 1D). Finally, the normal embryonic stem-like signature specifically harbors genes linked to both RNA metabolism and translation (Figure 1F).

**Figure 2:**
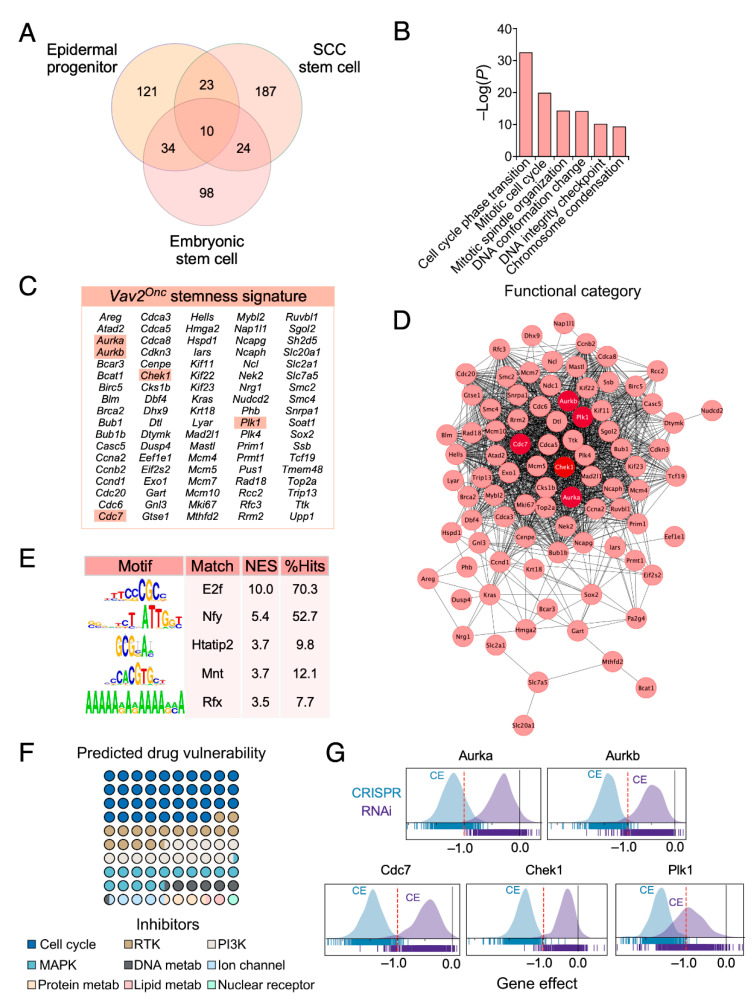
Identification of druggable Vav2^Onc^-induced cell stemness targets. (**A**) Venn diagram showing the overlap among the different Vav2^Onc^-induced stemness signatures according to the GSEA shown in Figure 1A,C,E. (**B**) Main functional categories encoded by the Vav2^Onc^ common SCL signature (CSS). (**C**) List of genes comprising the Vav2^Onc^ CSS. Those that were selected as candidate druggable targets are highlighted in color. (**D**) Protein interaction network of the genes comprising the Vav2^Onc^ CSS. Those that were selected as candidate druggable targets are highlighted in dark color. (**E**) Enriched transcription factor binding sites in the promoter regions of the genes belonging to the Vav2^Onc^ CSS. The normalized enrichment score (NES) and percentage of hits for each transcription factor binding site are also indicated. (**F**) Sector graph showing the top drug categories enriched against the Vav2^Onc^ CSS according to the LINCS project. The number of circles is proportional to the enrichment Z score. (**G**) DepMap analysis of the dependency of tumor cell line panels in CRISPR (blue) and RNAi (purple) databases for the indicated genes (top). Strong dependency is indicated as common essential (CE).

We next investigated whether this stem cell-like programs were associated with some “gene addiction” that could be exploited pharmacologically. In order to find the most promising targets, we first decided to analyze the genes that were redundantly present in at least two of the foregoing SCL transcriptional programs triggered by Vav2^Onc^. We found 91 genes (18% of the total number of genes) that fulfilled this *a priori* criterion (Figure 2A). This set of genes, referred hereafter as the Vav2^Onc^ common SCL signature (CSS), encodes proteins mainly involved in cell cycle progression such as cyclin family members, protein kinases, helicases, topoisomerases, nucleases, kinesins, and variety of transcription factors (Figure 2B–D).

Analyses of enriched transcription factor binding sites suggest that the expression of the Vav2^Onc^ CSS is under the control of the transcriptional factors E2F, NFY and, to a lesser extent, Htatip2, Mnt and Rfx (Figure 2E) [33,34,35,36,37].

We next used the library of integrated network-based cellular signatures to predict potential chemical perturbagen vulnerabilities derived from the Vav2^Onc^ CSS [24]. In keeping with the microarray and functional annotation data, we found that the highest-ranking group of drugs predicted to be effective against this Vav2^Onc^-driven transcriptional program includes inhibitors for cell cycle-associated proteins, receptor protein tyrosine kinases (RTKs), phosphatidylinositol 3-kinase (PI3K), and mitogen-activated protein kinases (MAPKs) (Figure 2F). These data are also consistent with previous studies indicating that the elimination of VAV proteins in the skin leads to defects in both Ras–ERK and PI3K–Akt signaling [30]. We next selected a number of potential pharmacological targets within the Vav2^Onc^-driven CSS using two a priori criteria: (i) Clear association with tumor cell fitness according to the Cancer Dependency Map Project [25] (Figure 2G). (ii) Availability of commercial inhibitors for them. This strategy led us to focus our analysis on five candidate targets: aurora kinases A (Aurka) and B (Aurkb), the cell division cycle 7-related protein kinase (Cdc7), the checkpoint kinase 1 (Chek1) and the polo-like kinase 1 (Plk1) (Figure 2C,D,G).

### 3.2. Keratinocytes with High Vav2 Activity are Highly Dependent on Aurora, Cdc7, Check1 and Plk1 Kinase Activities

**Figure 3:**
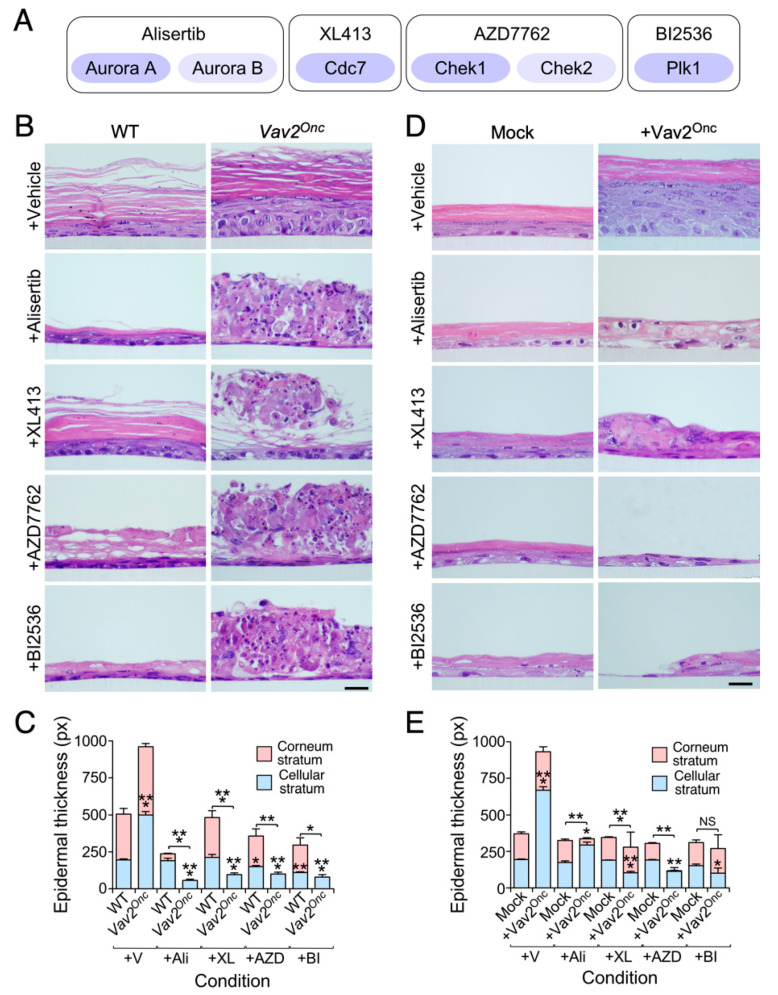
VAV2 upregulation leads to vulnerability against cell cycle inhibitors. (**A**) Scheme indicating the targets for each of the drugs used in this study. Primary and secondary targets are indicated in dark and light blue, respectively. (**B**) Representative hematoxylin-eosin tissue sections from organotypic cultures using the primary mouse keratinocytes of the indicated genotypes (top) upon treatment with the indicated drugs (left). Scale bar, 10 μm (*n* = 3). WT, wild-type. *Vav2*^Onc^; *Vav2*^Onc/Onc^ mice. (**C**) Quantification of the thickness of the indicated epidermal strata according to the data obtained in panel B. *, *p* < 0.05; **, *p* < 0.01; ***, *p* < 0.001 (ANOVA and Tukey’s HSD test, *n* as in panel B). V, vehicle; Ali, Alisertib; XL, XL413; AZD, AZD7762; BI, BI2536. (**D**) Representative hematoxylin-eosin tissue sections from organotypic cultures using the KerCT human keratinocytes expressing the indicated proteins (top) upon treatment with the indicated drugs (left). Scale bar, 10 μm (*n* = 3). (**E**) Quantification of the thickness of the indicated epidermal strata according to the data obtained in panel D. NS, not significant; *, *p* < 0.05; **, *p* < 0.01; ***, *p* < 0.001 (ANOVA and Tukey’s HSD test, *n* as in panel D). Abbreviations are as in C. In C and E, data represent the mean ± SEM.

To validate the targets identified in Figure 2, we next tested the effect of a collection of pharmacologic inhibitors for aurora kinases a and b (Alisertib), Cdc7 (XL413), Chek1 (AZD7762) and Plk1 (BI2536) (Figure 3A). All the selected compounds show high specificity towards the targets, with the exception of AZD7762 that can also block the related Chek2 kinase [38,39,40,41] (Figure 3A). These inhibitors were tested in 3D organotypic cultures of keratinocytes to mimic what happens in the context of a fully formed epithelium. In addition, this strategy allowed us to specifically address the effect of the inhibitors in the basal, suprabasal and fully differentiated keratinocyte layers present in this cell culture-generated epithelium. Importantly, we chose concentrations of each drug (10 nM) that did not cause in pilot experiments significant interference with the growth and differentiation of control cells under 3D culture conditions (Figure 3B,C). With this strategy, we expected to unveil vulnerabilities specifically linked to the gain-of-function events elicited by Vav2^Onc^ in those cells.

Under these culture conditions, the primary keratinocytes isolated from the skin of newborn *Vav2*^Onc/Onc^ mice form a hyperplastic epithelium (Figure 3B,C), thus recapitulating the phenotype observed in the skin and head and neck areas of *Vav2*^Onc/Onc^ mice [11]. The addition of each of those inhibitors to the 3D cultures triggers massive cell death in the suprabasal layers of the epithelium formed by *Vav2*^Onc/Onc^ keratinocytes (Figure 3B,C). However, the basal layers display a normal structure, forming a thin but viable epithelium (Figure 3B,C). The effect observed is specific for Vav2^Onc^-expressing suprabasal cells, because the inhibitors do not elicit any over effect on the epithelial structures formed by the keratinocytes obtained from the wild-type mice (Figure 3B,C).

To extend these findings to the human context, we next tested the effect of those drugs in 3D organotypic cultures of human keratinocytes (Ker-CT cells). These cells have been immortalized, but not transformed, by the ectopic expression of both human telomerase reverse transcriptase and cyclin-dependent kinase 4 [42]. As in the case of keratinocytes from newborn *Vav2*^Onc/Onc^ mice, we found that the human keratinocytes ectopically expressing the constitutively active mouse Vav2^Onc^ protein could also generate a highly hyperplastic epithelium (Figure 3D,E). The addition of Alisertib and XL413 blocks this hyperplastic phenotype, leading to the formation of epithelia with a thickness similar to than found in control cells (Figure 3D,E). The treatments with AZD7762 and BI2536 elicit a more dramatic effect, leading to the formation of aberrant epithelial structures that were even thinner than those generated by control cells (Figure 3D,E). The reason for the disparity in the effects of the inhibitors on mouse and human keratinocytes is unknown. It can be due to the much higher levels of expression of Vav2^Onc^ in the latter cells or, alternatively, to the fact that these cells also ectopically express the cyclin-dependent kinase 4.

### 3.3. The Viability of hnSCC Cells is Highly Dependent on Vav2^Onc^-Regulated Actionable Targets

**Figure 4:**
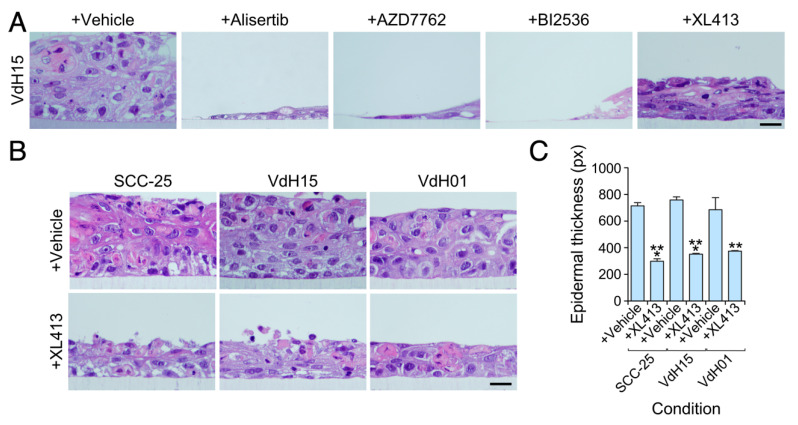
Cdc7 inhibitors impair the growth of head and neck cell squamous carcinoma patient-derived cells and cancer cell lines. (**A**) Representative hematoxylin-eosin tissue sections from organotypic cultures of VdH15 cells upon treatment with the indicated drugs (top). Scale bar, 10 μm (*n* = 3). (**B**) Representative hematoxylin-eosin sections from organotypic cultures using the indicated cell models (top) upon treatment with the XL413 compound (left). Scale bar, 10 μm (*n* = 3). (**C**) Quantification of the thickness of the epithelium according to the data obtained in panel B. **, *p* < 0.01; ***, *p* < 0.001 (Student’s *t*-test, *n* as in panel A). Data represent the mean ± SEM.

We have recently shown that endogenous wild-type human VAV2, which is overexpressed in many hnSCC samples and hnSCC patient-derived cells [11], is critical for the tumorigenic and metastatic properties of hnSCC cancer cell lines (SSC-25) and hnSCC patient-derived cells (VdH15, VdH01) when tested in orthotopic xenotransplant experiments in immunocompromised mice. This phenotype is associated with reduced and increased levels of cell proliferation and terminal differentiation, respectively [11]. When tested in 3D organotypic cultures, the *VAV2* knockdown cells exhibit the same proliferative and differentiation defects when compared to the respective parental SSC-25, VdH15 and VdH01 counterparts [11]. We observed that the addition of Alisertib, AZD7762 and BI2536 to those cells results in a significant destruction of the 3D cultures generated by each of those cell models (Figure 4A and data not shown). This indicates that the viability of these three types of hnSCC cells is highly dependent on the activity of aurora, Chek1, and Plk1 kinases. By contrast, we found that the XL413 elicited a milder effect that resulted in the reduced ability of these cells to form highly enlarged and deorganized tissues in 3D culture conditions (Figure 4A–C). Collectively, these results indicate that the activity of aurora, Cdc7, Check, and Plk1 are critical Achilles’ heels for the viability of keratinocytes and hnSCCs containing high levels of endogenous VAV2 activity. By contrast, the activity of each of those kinases is less relevant for naïve keratinocytes, even those that, as is the case of *Vav2*^Onc/Onc^ basal keratinocytes, normally exhibit high levels of proliferative activity.

### 3.4. The Vav2^Onc^ Stem Cell-Like Transcriptome Has Prognostic Value

**Figure 5:**
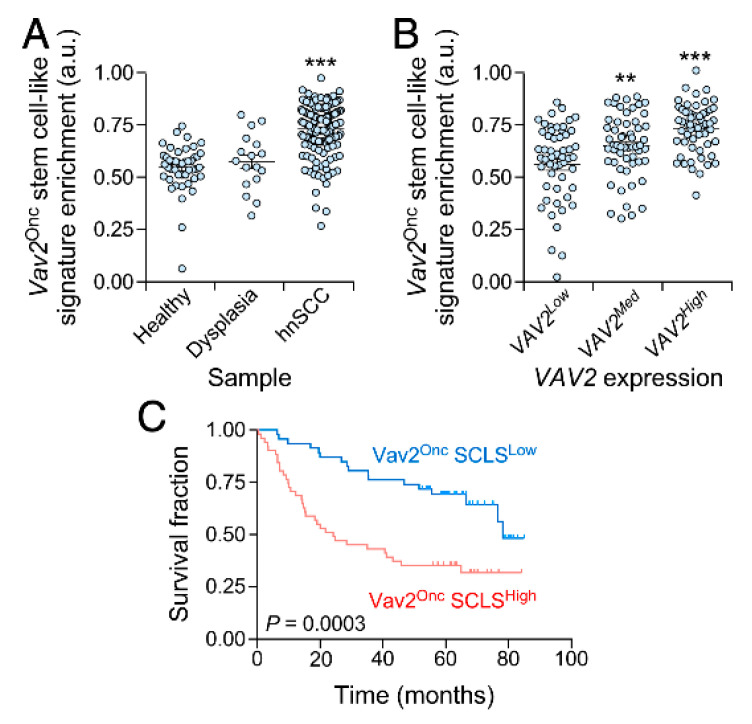
The Vav2^Onc^-induced stemness signature has prognostic value in head and neck squamous cell carcinoma patients. (**A**) Dot plot showing the enrichment of the Vav2^Onc^ SCL signature in the indicated samples from the GSE30784 dataset. ***, *p* < 0.001 (ANOVA and Tukey’s HSD test). hnSCC, head and neck squamous cell carcinoma. (**B**) Dot plot showing the enrichment of the Vav2^Onc^ SCL signature according to *VAV2* expression levels in tumor samples from the GSE30784 dataset. **, *p* < 0.01; ***, *p* < 0.001 (ANOVA and Tukey’s HSD test). (**C**) Kaplan-Meier survival plots of hnSCC patients from GSE41613 dataset according to the enrichment of the Vav2^Onc^ SCL signature. The Mantel–Cox test *p* value is indicated.

Finally, we decided to investigate whether the Vav2^Onc^-regulated SCL gene expression program could be used as a diagnostic marker for hnSCC patients. Using an in-silico approach, we observed that this gene expression signature is indeed enriched in hnSCCs when compared to both normal and dysplastic tissue (Figure 5A). We also found that the levels of this signature are directly proportional to the abundance of the *VAV2* transcript in hnSCCs (Figure 5B), further linking this SCL program to the presence of upregulated VAV2 activity found in this type of tumors. More importantly, we observed that the Vav2^Onc^ SCL signature is associated with poor hnSCC patient prognosis (Figure 5C).

## 4. Discussion

**Figure 6:**
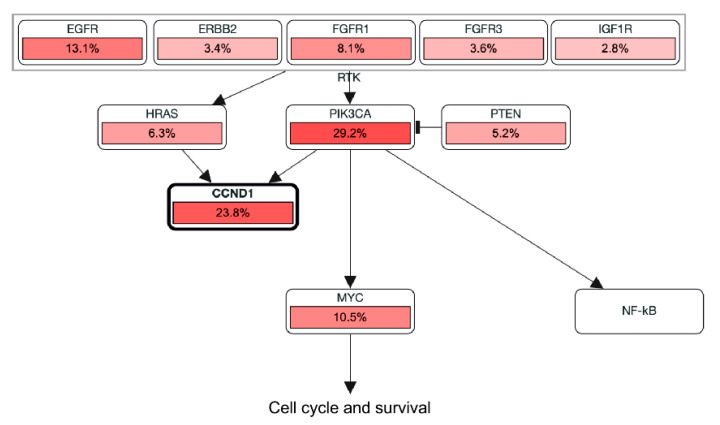
Head and neck squamous cell carcinoma pathways related to the Vav2^Onc^ CSS according to PathwayMapper. The frequency of oncogenic lesions found in each gene are indicated.

Cancer cells arise as a consequence of the pervasion of the same molecular pathways that are employed by stem and progenitor cells to both self-perpetuate and (re)generate tissues [1]. Such a phenomenon makes it challenging to pharmacologically target these pathways without harming the integrity of healthy tissue [1]. However, this is also a double-edged sword for cancer cells since by creating these specific molecular perturbations they usually become addicted to them [4]. This constitutes a therapeutic opportunity that can be exploited by identifying biomarkers able to predict such cancer addiction to specific molecular programs. Here we have shown that the upregulation of the activity of the GEF VAV2 in keratinocytes leads to the upregulation of a large number of genes associated with both stem cell-like and cell cycle features. Interestingly, PathwayMapper [43] analyses show that the Vav2^Onc^ stem cell-like signature is closely related to the presence of oncogenic alterations in key loci such as *EGFR*, *FGFR1*, *HRAS*, *PIK3CA*, c-*MYC*, and *CCND1* loci in hnSCC (Figure 6). Importantly, we have also shown that this Vav2^Onc^-regulated gene expression program has a number of pharmacological Achilles’ heels, including aurora kinase family members, Check1, Cdc7 and Plk1.

Interestingly, we have found that the Cdc7 inhibitor XL413 shows the best effectiveness/toxicity ratio in all three 3D organotypic models tested in our study. This is consistent with previous studies indicating that this drug is well tolerated by healthy cells [44,45]. In the SCC context, it has been demonstrated using both in vitro assays and xenograft models that this inhibitor holds great therapeutic promise [45]. According to our data, this therapeutic response will probably be stronger in cancer cells exhibiting high levels of VAV2 activity.

## 5. Conclusions

Collectively, these data show that the association between VAV2 and the induction of stem cell-like transcriptional programs has translational implications that can be used to identify druggable opportunities in the SCC context. The identification of inhibitors able to counteract excessive VAV2 signaling from a distal downstream position is of special interest, given that the inhibition of the catalytic activity of most RHO GEFs has been difficult to achieve up to now using pharmacological approaches [6]. This is due to the shallow nature of the GEF–RHO GTPase catalytic interface [6]. This underscores the value of implementing multifaceted strategies like the one presented here towards the identification of pharmacologically actionable downstream targets and programs. In this regard, the development of new strategies based on high-throughput biological data and next-generation deep learning bioinformatic algorithms [46] will probably lead the way in the discovery of a new repertoire of cancer vulnerabilities.
